# Increasing Safety Plan Use and Reducing Suicidal Ideation Among Emerging Adults: A Pilot Randomized Trial of the STARS Intervention

**DOI:** 10.1111/sltb.70101

**Published:** 2026-04-17

**Authors:** Lily A. Brown, Jennifer T. Tran, Robert Gallop, Jessica L. Webster, James R. Wolfe, Marin M. Kautz, Yiqin Zhu, Amanda Arcomano, Jennifer Ben Nathan, Lindiwe Mayinja, Alexander Azat O'Connor, Danielle Mowery, David S. Mandell, Gregory K. Brown, Maria A. Oquendo, José A. Bauermeister

**Affiliations:** ^1^ Department of Psychiatry Perelman School of Medicine, University of Pennsylvania Philadelphia Pennsylvania USA; ^2^ Department of Family & Community Health School of Nursing, University of Pennsylvania Philadelphia Pennsylvania USA; ^3^ Department of Mathematics West Chester University Pennsylvania USA

**Keywords:** eHealth, emerging adults, LGBTQ+, mental health, suicide prevention

## Abstract

**Introduction:**

Emerging adult sexual and gender minorities (EA‐SGM) experience disproportionately high rates of suicide. The Safety Planning Intervention can reduce suicide risk, but its effectiveness for this population may be limited without additional support. The Supporting Transitions to Adulthood to Reduce Suicide (STARS) program was developed to address this gap by integrating a mobile application and peer mentorship to promote consistent Safety Plan use.

**Methods:**

Participants (*n* = 64) were randomized to receive either SPI alone or SPI plus STARS. Participants were followed for 6 months.

**Results:**

STARS was highly acceptable and associated with significantly greater use of the Safety Plan at 2 months compared to SPI alone. While both groups demonstrated significant reductions in suicidal ideation over time, participants in STARS showed sustained nonsignificant improvements through 6 months, whereas SPI alone experienced a slight increase after 4 months. STARS participants used their Safety Plan significantly more frequently than SPI alone (39% vs. 15% for monthly use) at 2 months.

**Conclusions:**

STARS is a feasible and acceptable intervention that enhances Safety Plan engagement and longer‐term reductions in suicidal ideation among EA‐SGM. These promising findings provide preliminary support for a fully powered effectiveness trial to evaluate STARS' outcomes.

**Trial Registration:**

ClinicalTrials.gov identifier: NCT 05018143

## 1 Introduction

Emerging adult sexual and gender minorities (EA‐SGM), aged 18–24, experience more suicidal ideation and behaviors than their heterosexual and cisgender peers (Kann 2018; Rogers and Taliaferro 2020). EA‐SGM also experience unique risk factors for suicide given increased risk of isolation from family, reduced positive affect, and increased experiences of discrimination compared to their heterosexual and cisgender peers (Green et al. 2021; Robinson 2018). Effects of these risk factors may be especially pronounced as youth transition from adolescence into adulthood and experience new psychosocial stressors and pressures as a result (Hatchel et al. 2021). Suicide is the second leading cause of death in this age group in the United States (Centers for Disease Control and Prevention 2024). In nationally representative data, sexual minority emerging adults had significantly higher adjusted odds of reporting a suicide plan compared to their heterosexual peers, with adjusted odds ratios ranging from approximately two‐ to six‐fold depending on subgroup and age stratum (Ramchand et al. 2022). EA‐SGM require unique considerations for suicide prevention based both on their developmental window (Arnett 2000) and identity (Ramchand et al. 2022). Despite these alarming statistics, few evidence‐based suicide interventions have been tailored to address the unique challenges that EA‐SGM face.

The Safety Planning Intervention (SPI; Stanley and Brown 2012) is an evidence‐based approach to reduce risk of suicide attempts (Stanley et al. 2018). SPI involves collaboratively developing a personalized Safety Plan with individuals at risk of suicide. This plan identifies early warning signs, internal coping strategies, and sources of social and professional support. Effectiveness of SPI on suicidal behavior outcomes has been demonstrated as a standalone intervention (Boudreaux et al. 2023; Hasking et al. 2024) and when combined with follow‐up services (Stanley et al. 2018; Weinstock et al. 2025). While SPI is effective across various clinical contexts, it faces barriers to sustained use. Many individuals struggle to access or engage with their Safety Plan in crisis, citing practical challenges (e.g., losing the paper form containing the written Safety Plan) and psychosocial barriers (e.g., lack of motivation during crises; isolation; Kayman et al. 2015). While the SPI was originally developed for managing acute suicidal crises, clinicians commonly report using the intervention with a wide‐range of clinical presentations involving suicidal ideation in their practices (Moscardini et al. 2020).

Mobile technology offers promising avenues to improve SPI delivery. Digital platforms can increase accessibility and enhance usability by making Safety Plans interactive, editable, and available on‐demand. Yet even the most well‐designed mobile applications may fall short if they cannot sustain user engagement—especially in marginalized communities, where technology fatigue, distrust, and limited personalization can reduce long‐term use (Kidman et al. 2024). Incorporating peer mentoring into a digital intervention framework offers a complementary approach to overcome this limitation (Kaufman et al. 2022). Peer mentors, who share lived experiences with EA‐SGM, can provide culturally relevant support, foster accountability, and serve as navigators to help participants overcome barriers to using their Safety Plan. This combination of digital and interpersonal support may address the logistical and emotional barriers to Safety Plan use.

We in the Department of Psychiatry and the School of Nursing at the University of Pennsylvania developed the “Supporting Transitions to Adulthood and Reducing Suicide” (STARS) program as a hybrid intervention to support EA‐SGM at risk of suicide (Brown et al. 2023). STARS integrates an editable, app‐based Safety Plan with peer mentoring. Together, these components aim to address the unique needs of EA‐SGM by targeting core mechanisms of suicidal risk for the population (e.g., positive affect, social support, coping with discrimination, etc.) based on prior literature that demonstrated the importance of each of these factors (for examples, see Busby et al. 2020; de Lange et al. 2022; Mereish et al. 2023). This manuscript reports findings from a pilot randomized controlled hybrid type 1 implementation‐effectiveness trial testing the preliminary efficacy and acceptability of the STARS intervention. Participants were randomized to receive either SPI alone or SPI plus STARS (intervention), with outcomes tracked over 6 months. Primary outcomes included feasibility, acceptability, and changes in suicidal ideation and behavior; secondary outcomes included app engagement and Safety Plan use. We hypothesized that participants assigned to STARS would report higher rates of Safety Plan use and greater reductions in suicidal ideation compared to the SPI alone group. Additionally, we hypothesized that, within the intervention condition, greater engagement with STARS, defined by more frequent logins to the STARS app, would be associated with greater reductions in suicidal ideation.

## 2 Materials & Methods

### 2.1 Study Design

**FIGURE 1:**
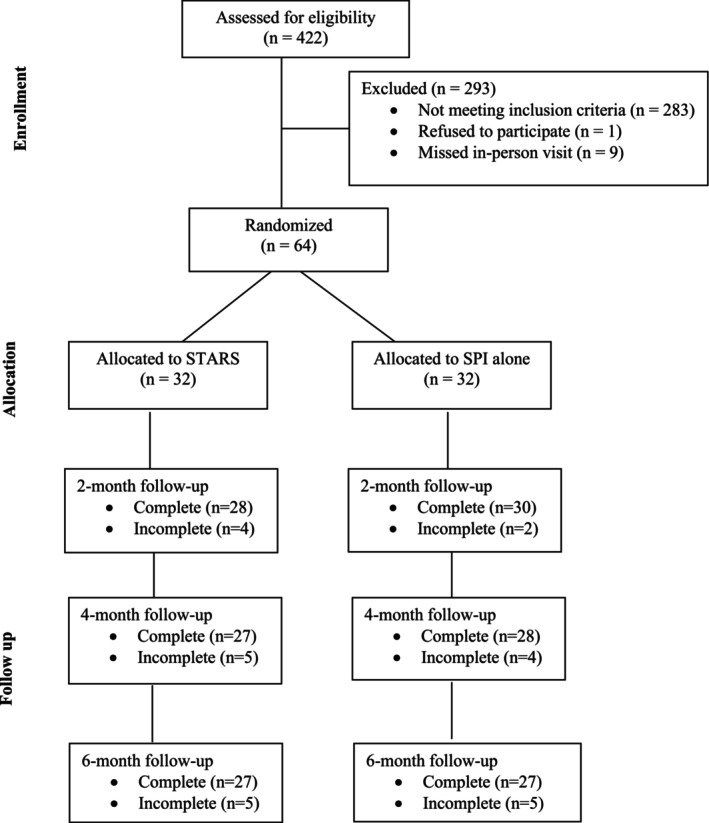
Consort diagram.

This pilot randomized controlled trial evaluated the acceptability and initial efficacy of the STARS application across 6 months (baseline, 2‐month, 4‐month, and 6‐month follow‐up). Participants were randomly assigned in a 1:1 ratio to either the STARS intervention (SPI + STARS app + peer mentoring) or control arm (SPI only). This study is reported in accordance with the CONSORT 2010 guidelines for pilot and feasibility trials (Figure 1).

### 2.2 Ethics Approval

The University of Pennsylvania Institutional Review Board reviewed and approved the study protocol (849500), registered on ClinicalTrials.gov (NCT 05018143).

### 2.3 Participants and Recruitment

Recruitment occurred between October 2022 and January 2024 via social media and flyers in the Philadelphia area. Eligibility criteria included: (1) aged 18–24 years; (2) sexual or gender minority identity; (3) residence in Greater Philadelphia; (4) smartphone and Wi‐Fi access; (5) plans to remain in the area for the next 6 months; (6) no self‐reported psychosis; and (7) past‐month suicidal ideation. Table 1 describes demographic and clinical features of the sample.

**TABLE 1:** Demographic characteristics of participants by randomization.

Demographic variable	Total	Group		Test	Test statistic	*p*
		SPI alone	STARS			
*N*	64	32	32	—	—	—
Age (SD)	21.41 (1.83)	21.81 (1.60)	21.00 (1.98)	*t*‐test	1.81	0.76
Race (%)						
Asian American	18 (28.1)	10 (31.3)	8 (25.0)	*χ* ^2^	1.71	0.79
Black or African American	7 (10.9)	4 (12.5)	3 (9.4)			
Multiracial	5 (7.8)	3 (9.4)	2 (6.3)			
White	32 (50.3)	14 (43.7)	18 (56.3)			
Unknown	2 (3.1)	1 (3.1)	1 (3.1)			
Ethnicity (%)						
Hispanic	9 (14.1)	6 (18.8)	3 (9.4)	*χ* ^2^	1.16	0.28
Not Hispanic	55 (86.9)	26 (81.2)	29 (90.6)			
Race/Ethnicity (NIH)						
NH AI/AN	0 (0)	0 (0)	0 (0)	*χ* ^2^	7.87	0.35
Hispanic AI/AN	0 (0)	0 (0)	0 (0)			
NH Asian American	18 (28.1)	10 (31.3)	8 (25.0)			
Hispanic Asian American	0 (0)	0 (0)	0 (0)			
NH Black	4 (6.3)	1 (3.1)	3 (9.4)			
Hispanic Black	3 (5.0)	3 (9.4)	0 (0)			
NH White	29 (45.3)	13 (40.6)	16 (50)			
Hispanic White	3 (5.0)	1 (3.1)	2 (6.3)			
NH multiracial	4 (6.3)	2 (6.3)	2 (6.3)			
Hispanic multiracial	1 (1.6)	1 (3.1)	0 (0)			
NH unknown	0 (0)	0 (0)	0 (0)			
Hispanic unknown	2 (3.1)	1 (3.1)	1 (3.1)			
Sex assigned at birth (%)						
Female	48 (75.0)	27 (84.4)	21 (65.6)	*χ* ^2^	1.16	0.76
Male	13 (20.3)	5 (15.6)	8 (25.0)			
Third gender	1 (1.6)	0 (0)	1 (3.1)			
Prefer not to answer	2 (3.1)	1 (3.1)	1 (3.1)			
Gender identity^a^						
Agender	2 (3.1)	0 (0)	2 (6.3)	*χ* ^2^	11.15	0.13
Cisgender man	11 (17.2)	2 (6.3)	9 (28.1)			
Cisgender woman	31 (48.4)	17 (53.1)	14 (43.8)			
Genderqueer	9 (14.1)	8 (25.0)	1 (3.1)			
Nonbinary	16 (25)	7 (21.9)	9 (28.1)			
Questioning	5 (7.8)	2 (6.3)	3 (9.4)			
Transgender man	7 (10.9)	3 (9.4)	4 (12.5)			
Transgender woman	3 (5.0)	2 (6.3)	1 (3.1)			
Sexual identity						
Asexual	2 (3.1)	1 (3.1)	1 (3.1)	*χ* ^2^	2.71	0.91
Bisexual	28 (43.8)	16 (50)	12 (37.5)			
Gay	12 (18.8)	5 (15.6)	7 (21.9)			
Lesbian	11 (17.2)	5 (15.6)	6 (18.8)			
Queer	7 (10.9)	4 (12.5)	3 (9.4)			
Pansexual	2 (3.1)	1 (3.1)	1 (3.1)			
No label	1 (1.6)	0 (0)	1 (3.1)			
Questioning	1 (1.6)	1 (3.1)	0 (0)			
Highest level of education (%)						
9th–11th grade	1 (1.6)	1 (3.1)	0 (0)	*χ* ^2^	3.77	0.71
High school diploma	14 (21.9)	5 (15.6)	9 (28.1)			
Some college	23 (35.9)	12 (37.5)	11 (34.4)			
Technical school graduate	1 (1.6)	1 (3.1)	0 (0)			
Four‐year college graduate	21 (32.8)	11 (34.4)	10 (31.3)			
Some graduate school	3 (4.7)	2 (6.3)	1 (3.1)			
Master's degree or above	1 (1.6)	0 (0)	1 (3.1)			
Employment status (%)						
Full‐time work	13 (20.3)	5 (15.6)	8 (25.0)	*χ* ^2^	3.58	0.61
Part‐time work	25 (39.1)	13 (40.6)	12 (37.5)			
Unemployed, but looking	14 (21.9)	7 (21.9)	7 (21.9)			
Unemployed, not looking	6 (9.4)	4 (12.5)	2 (6.3)			
Another status	3 (4.7)	2 (6.3)	1 (3.1)			
Prefer not to answer	3 (4.7)	2 (6.3)	1 (3.1)			
Income						
Less than $10,999	38 (59.4)	16 (50.0)	22 (68.8)	*χ* ^2^	4.20	0.52
$11,000‐19,999	8 (12.5)	4 (12.5)	4 (12.5)			
$20,000–$29,999	8 (12.5)	6 (18.8)	2 (6.3)			
$30,000–$39,999	3 (4.7)	1 (3.1)	2 (6.3)			
$40,000–$49,999	2 (3.1)	1 (3.1)	1 (3.1)			
Prefer not to answer	5 (7.8)	1 (3.1)	4 (12.5)			
Insurance						
Yes, I have my own	19 (29.7)	10 (31.3)	9 (28.1)	*χ* ^2^	1.41	0.84
Yes, I am covered by parents	39 (60.9)	19 (59.4)	20 (62.5)			
No	3 (4.7)	2 (6.3)	1 (3.1)			
I don't know	1 (1.6)	0 (0)	1 (3.1)			
Prefer not to answer	2 (3.1)	1 (3.1)	1 (3.1)			
Living alone						
Yes	12 (18.8)	3 (9.4)	9 (28.1)	*χ* ^2^	3.69	0.06
No	52 (81.3)	29 (90.6)	23 (71.9)			
Gender affirming care						
Yes	2 (3.1)	1 (3.1)	1 (3.1)	*χ* ^2^	0	1.00
No	62 (96.9)	31 (96.9)	31 (96.9)			
Medical treatment past year						
Yes	59 (92.2)	27 (84.4)	32 (100)	*χ* ^2^	5.42	0.07
No	4 (6.3)	4 (12.5)	0 (0)			
Prefer not to answer	1 (1.6)	1 (3.1)	0 (0)			
Lifetime suicide attempt						
0	36 (56.3)	21 (65.6)	15 (46.9)	*χ* ^2^	2.29	0.13
1	14 (21.9)	5 (15.6)	9 (28.1)			
More than 1	14 (21.9)	6 (18.8)	8 (25.0)			
Lifetime suicidal ideation						
No	0 (0)	0 (0)	0 (0)	—	—	—
Yes	64 (100)	32 (100)	32 (100)			

^a^ Participants can select more than one answer; therefore, numbers may not add up to the total *N*. No differences between groups.

Following Ahn et al.'s (2001) power formula, a target sample size was selected to allow for the detection of intervention differences over the four repeated time points. These power calculations are based on a linear mixed models approach with the following assumptions: an expected sample size of 29 per group, a within‐subject correlation of 0.5, 5% attrition at each time point, a 2‐sided test, and a Type I‐error level of 0.05. The within‐subject correlation and missing data rate are based on Pistorello et al. (2017) which saw a within‐subject correlation of 0.47 and an attrition rate of 7% at each timepoint. While the Pistorello et al. (2017) study reported higher levels of baseline ideation, the age group of the participants is comparable. Based on these assumptions, we cross 80% power to detect a medium between‐group effect size on suicidal ideation (Cohen *d* = 0.60). We ultimately recruited 32 participants per group, increasing power from 80.4% to 84.1% based on the original assumptions. As a pilot trial, this study was not powered to detect small effects but was instead designed to examine its feasibility, acceptability, and preliminary efficacy.

### 2.4 Study Procedures

Interested individuals were directed to complete a brief online screener. Eligible individuals completed a phone‐based risk assessment and received information about the study. At an in‐person baseline visit, participants completed informed consent, were assessed by a licensed clinician using the Columbia–Suicide Severity Rating Scale (C‐SSRS; Posner et al. 2011), and received a SPI. They were then randomized using REDCap's computer‐generated sequence. Due to the nature of the intervention, participants and study staff were not blinded; however, evaluators and analysts were masked.

Participants received $50 at baseline via ClinCard, a reloadable debit card. Follow‐up assessments at 2, 4, and 6 months were completed online via a personalized REDCap link and compensated at $30, $40, and $50, respectively.

### 2.5 Intervention Descriptions

#### 2.5.1 SPI

All participants completed an in‐person Safety Plan Intervention, an evidence‐based suicide prevention practice. SPI empowers individuals to identify and use personal and external resources to mitigate distress and prevent suicidal behavior. Following a narrative discussion of the individual participant's recent suicide crisis and rationale for the safety plan using the suicide risk curve, the clinician supported the participant in building a personalized Safety Plan. The Safety Plan consists of six clinician‐guided steps: (1) Identifying Warning Signs (i.e., early recognition of distress cues); (2) Internal Coping Strategies (i.e., simple, self‐directed distraction techniques); (3) Social Contacts for Distraction (i.e., identifying supportive people or settings); (4) Reaching Out for Support (i.e., listed trusted individuals for emotional support); (5) Professional Resources (i.e., customized referrals) (e.g., hotlines, therapists); and (6) Lethal Means Safety (i.e., reducing access to environmental risks). The SPI was completed on paper, with the participant and the clinician writing the Safety Plan down separately, though participants were coached to decide how they want to access the plan when they need it in the future (i.e., on paper, transform it into a digital copy using a method of their preference, take a picture of it, etc.).

#### 2.5.2 STARS

Participants randomized to the STARS arm received 6‐month access to a mobile app, downloaded at the baseline evaluation following randomization, and six peer mentoring sessions. The STARS app features a digital, editable version of the participant's SPI which was uploaded to the app once they were randomized. Safety Plans included tappable, auto‐dial phone numbers, and an emergency “Call 911” button for real‐time access. Participants could modify their plan as needed. In addition to the Safety Plan, the app included goal‐setting tools, gamification elements (e.g., badges, points), a moderated peer forum, and multimedia educational content and interactive activities focused on identity, relationships, emotional well‐being, and life skills.

Peer mentoring sessions were scheduled to occur weekly over approximately 6 weeks. Each 30‐min virtual session was facilitated by a trained peer mentor and focused on reinforcing Safety Plan use, and addressed a weekly topic (e.g., addressing discrimination, enhancing positive affect). Session 1 focused on reinforcing Safety Plan use, session 2 on values clarification and goal setting, session 3 on dealing with negative self‐talk, session 4 on behavioral activation, session 5 on coping with discrimination, and session 6 on building close relationships. STARS app content reinforced this session content and offered additional content on mental health, developmental transitions (healthy dating, applying for college and jobs, etc.) and connecting to safe spaces that affirm identity. All six peer mentorship sessions were scheduled in the app at the baseline evaluation. Participants could track past and upcoming peer mentor sessions as well as reschedule them within the app. Full details on peer mentor selection, training, supervision and fidelity are reported elsewhere (Tran et al. 2025); in brief, peer mentors were selected based on a self‐reported interest in serving as a peer mentor to support suicide prevention among the LGBTQIA+ emerging adult population. Fidelity was ensured through standardized peer mentor training in peer mentorship, suicide risk, and principals of cognitive behavioral therapy by a counselor and psychologist (16 h initially followed by refreshers throughout, with weekly consultation) and observation of 15% of sessions by licensed clinicians (for more details, see Tran et al. 2025).

At the beginning of each session, peer mentors engaged participants in structured questions to review their use of the Safety Plan. This included checking whether participants had used their Safety Plans, discussing any obstacles encountered, and brainstorming strategies to address these challenges. Mentors also reminded participants of the Safety Plan's steps and explored potential modifications to improve its relevance and usability. By encouraging regular Safety Plan use, the sessions aimed to reduce barriers that might prevent users from seeking help during a crisis. Through these sessions, the STARS intervention created a structured yet adaptable platform for providing emotional and practical support, empowering participants to implement their Safety Plans effectively while fostering a sense of belonging and resilience.

## 3 Measures

Primary outcomes were acceptability and suicidal ideation. Secondary outcomes included Safety Plan use and app engagement.

### 3.1 Sociodemographic Characteristics

Age, race/ethnicity, sex assigned at birth and gender identity, sexual orientation, insurance, socioeconomic status, mental health history, and lifetime suicide attempt were assessed at baseline (Table 1).

#### 3.1.1 Feasibility

Feasibility of STARS was determined by the number of people who were eligible for a baseline evaluation based on the phone screen, the number who attended the baseline evaluation, the number who enrolled, and the number of peer mentor sessions completed among those who were randomized to STARS.

### 3.2 Acceptability

Participants assigned to STARS completed a postintervention assessment of usability and acceptability at 2‐, 4‐, and 6‐month follow‐ups. Items were theory‐informed and adapted from established digital health and technology acceptance frameworks, including the System Usability Scale (SUS; Brooke 1996), the Technology Acceptance Model (TAM; Venkatesh and Davis 2000), and mobile health usability instruments (Zhou et al. 2019). Adaptations were made to reflect specific STARS features (e.g., essential content, goals feature, system responsiveness, and navigation). Items assessed perceived ease of use (e.g., ease of navigation), system performance (e.g., loading speed), perceived usefulness (e.g., impact on managing life challenges), self‐efficacy for using core features, and intention to continue use and recommend the app.

Our items assessed four related domains: (1) perceived ease of use (e.g., navigation), (2) system performance (e.g., loading speed), (3) perceived usefulness and empowerment (e.g., impact on managing life challenges), and (4) behavioral intention and engagement, including confidence in using core features, likelihood of continued use, and willingness to recommend STARS to peers. Most items were rated on a 4‐point Likert‐type scale ranging from 1 (“strongly disagree”) to 4 (“strongly agree”). Likelihood of continued use was assessed on a 5‐point scale ranging from 1 (“very unlikely”) to 5 (“very likely”). Higher scores reflected greater usability and acceptability (Table 2).

**TABLE 2:** Intervention Acceptability among participants assigned to the STARS condition.

	2 months	4 months	6 months
	Mean (SD)	Mean (SD)	Mean (SD)
It was easy to download and install the app.	3.74 (0.53)	3.65 (0.49)	3.58 (0.50)
STARS loads all the text and graphics quickly.	3.37 (0.63)	3.35 (0.56)	3.31 (0.55)
STARS is easy to use.	3.39 (0.57)	3.41 (0.50)	3.37 (0.57)
STARS responds quickly when I click on a link or button.	3.21 (0.69)	3.37 (0.57)	3.33 (0.56)
It is easy to go back and forth between pages on STARS.	3.37 (0.63)	3.30 (0.61)	3.30 (0.61)
I find STARS is useful in my life.	3.15 (0.37)	2.96 (0.54)	2.91 (0.75)
I feel like I understand myself better since I started using STARS.	3.09 (0.53)	3.17 (0.70)	3.14 (0.66)
Using STARS enhances my effectiveness in dealing with life's challenges.	3.04 (0.45)	2.77 (0.77)	2.92 (0.70)
Using STARS makes it easier to live a healthier life.	2.94 (0.56)	2.73 (0.77)	2.95 (0.65)
I feel confident using the Essentials content within STARS to manage my wellbeing.	2.94 (0.64)	2.86 (0.66)	2.96 (0.61)
I feel confident using the Goals feature in STARS to set future goals.	2.95 (0.67)	2.68 (0.72)	2.79 (0.72)
I would recommend STARS to my friends.	3.15 (0.54)	3.22 (0.58)	3.15 (0.60)
*How likely would you be to continue using STARS if it were available?	3.79 (0.83)	3.63 (1.11)	3.52 (1.01)
I feel confident talking to a STARS Peer Mentor to discuss what's going on in my life.	3.54 (0.64)	3.36 (0.57)	3.58 (0.50)
Peer Mentor sessions were offered at times that worked for my schedule.	3.74 (0.53)	3.73 (0.45)	3.68 (0.69)
I was willing to download the app to meet with a Peer Mentor.	3.72 (0.54)	3.70 (0.47)	3.77 (0.43)
It was easy to use the app to talk with Peer Mentors.	3.52 (0.59)	3.52 (0.68)	3.45 (0.60)

*Note:* Items were answered on a 4‐point scale (1 = Strongly Disagree; 4 = Strongly Agree) except item labeled *, which is answered on a 5‐point scale (1 = Very Unlikely; 5 = Very Likely).

### 3.3 Safety Plan Use

At each follow‐up, participants were asked “Over the past two months, how often did you use your Safety Plan?” with the following response options: 1, Never; 2, Once or Twice; 3, Monthly; 4, Weekly; 5, Daily or Almost Daily; 999, Prefer not to answer.

### 3.4 Suicidal Ideation and Behavior

We employed two measures to capture both dimensional and categorical aspects of suicidal ideation. The first was the self‐report Beck Suicidal Ideation Scale (BSS; Beck and Steer 1991), which includes 21 items scored on a 0–3 scale. Higher scores from the sum of the first 19 items indicate greater severity of suicidal ideation. The BSS had good reliability in our sample (Cronbach's α range: 0.79–0.86 per assessment).

The second measure used was the interview‐rated severity of suicidal ideation subscale of the Columbia‐Suicide Severity Ratings Scale (C‐SSRS; Posner et al. 2011). The subscale includes five items with scores ranging from 0 to 5 with higher scores indicating greater severity of suicidal ideation. The intensity of ideation subscale includes 5 items, each rated on a scale from either 0–5 or 1–5 reflecting the frequency, duration, controllability, and reasons for suicidal ideation. Suicidal behavior was measured using the suicidal behavior subscale of the C‐SSRS to assess actual suicide attempts, interrupted and aborted suicide attempts, and preparatory behavior (Posner et al. 2011). For this measure, kappa coefficients for suicidal ideation items often exceed 0.85, indicating good interrater reliability (Posner et al. 2011).

#### 3.4.1 Engagement With STARS App

We extracted logins and Safety Plan clicks as behavioral engagement metrics (i.e., paradata; automatically captured data of users' app actions) recorded by the platform (Choi et al. 2023). These metrics served as proxies for overall engagement and direct interaction with the core intervention component.

### 3.5 Statistical Analysis Plan

We used descriptive statistics to summarize eligibility, enrollment, and retention (Figure 1). Baseline characteristics (e.g., age, gender identity, race/ethnicity) were described using means and standard deviations for continuous variables and frequencies for categorical variables. Independent‐sample *t*‐tests and chi‐square tests assessed balance across trial arms.

To examine intervention feasibility and preliminary signals of promise, we employed both within‐ and between‐group comparisons over time. We employed ordinal logistic regression models comparing the likelihood of more frequent use of the Safety Plan in STARS versus SPI alone (Scott et al. 1997). Repeated‐measures models assessed within‐ and between‐group trends over time. To assess intervention effects, statistical contrasts focused on differences over the entire longitudinal period, as well as contrasts at each assessment to better understand durability and/or timing of the intervention effect. For dichotomized outcomes (e.g., monthly use or greater), we used logistic regression and chi‐square tests at each time point (Kuritz et al. 1988).

Changes in suicidal ideation were analyzed using linear mixed‐effects models (for BSS scores) and generalized linear mixed models with a logit link (for C‐SSRS binary indicators) (Molenberghs and Verbeke 2006). We used an unstructured variance–covariance matrix (Verbeke and Molenberghs 2000) and the Kenward‐Roger approximation (Kenward and Roger 1997) to account for heterogeneity and degrees of freedom. Cohen's *d* was used to estimate within‐ and between‐group effect sizes following Feingold's method (2009). We also included a sensitivity analysis to further explore potential differences based on those with active suicidal ideation or higher vs. lower, given that the SPI is often implemented in more severe samples in research settings, but is implemented across a wider spectrum of risk in clinical settings.

To assess STARS engagement, we summarized app login frequency and Safety Plan access data. Associations between app use and suicidal ideation were explored using Pearson correlations adjusted for baseline ideation scores. Due to sample size limitations, mediation analyses were not conducted (Ten Have and Joffe 2012; VanderWeele 2016).

All analyses were intent‐to‐treat (ITT); therefore, all randomized participants are included in the analysis. As a sensitivity analysis for missing data (~12% across timepoints), Markov Chain Monte Carlo (MCMC) multiple imputation with 50 datasets via PROC MI in SAS 9.4 (Yuan 2011) was conducted on the average of the imputed data. This approach produces valid inferences when the imputation model includes all observed predictors (Rubin 1987).

We report point estimates and *p*‐values, including nonsignificant ones, to inform future trials. Per Leon et al. (2011), pilot studies evaluate feasibility and signal to justify or refine future testing. Following Kraemer et al. (2006), we do not use these results for power estimation or claims of efficacy, but include them to provide a transparent view of the intervention's potential promise, directionality, and variance structure as critical inputs for planning subsequent trials. Interpretations of marginal or null findings are offered cautiously, as part of the exploratory and iterative function of early‐phase research.

## 4 Results

### 4.1 Feasibility

The STARS study screened and enrolled participants between October 14, 2022, and January 26, 2024, with a total of 422 individuals assessed for eligibility (see Figure 1). Of these, 283 individuals were deemed ineligible because they did not meet the age requirement (*n* = 23), lived outside of Philadelphia (*n* = 60), planned to move within 6 months (*n* = 14), did not identify as SGM (*n* = 22), lacked a smartphone (*n* = 1), self‐reported psychosis (*n* = 100), or had not experienced suicidal ideation within the past month (*n* = 63). Thus, for the first metric of feasibility, 33% of all participants who responded to an online screen were eligible to be scheduled for a phone screen.

Among the 139 eligible participants, 57 could not be contacted, and 8 declined participation during the phone screening. This left 74 individuals who were enrolled for the baseline assessment. For the second metric of feasibility, 53% of those who completed a phone screen were eligible and scheduled for a baseline evaluation. Of these, 9 participants missed their baseline appointment and could not be recontacted; 1 participant declined to continue at this stage. For the third metric of feasibility, 86% of those scheduled for a baseline evaluation attended the evaluation and were enrolled in the study. Ultimately, 64 participants completed the baseline assessment and were randomized into either the STARS intervention group (*n* = 32) or the SPI alone group (*n* = 32). No adverse events or unintended effects related to the intervention were reported during the study. For our fourth metric of feasibility, 26 (81%) of those randomized to the STARS group completed all 6 of their peer mentor sessions and across all participants, 90% of all peer mentor sessions were completed. One participant missed only session 6, two participants missed sessions 5 and 6, two participants completed only 1 peer mentor session, and 1 participant opted out of peer mentorship sessions.

### 4.2 Sample Description

The study analyzed the demographic characteristics of 64 participants. The mean age was similar across groups (SPI alone: 21.81 ± 1.60 years; STARS: 21.00 ± 1.98 years). Participants primarily identified as non‐Hispanic White (*n* = 29, 45.3%), followed by non‐Hispanic Asian American (*n* = 18, 28.1%), non‐Hispanic Black (*n* = 4, 6.3%), and non‐Hispanic Multiracial (*n* = 4, 6.3%). Hispanic participants accounted for 14.1% of the sample (*n* = 9), including those who identified as Hispanic Black (*n* = 3, 5.0%), Hispanic White (*n* = 3, 5.0%), Hispanic Multiracial (*n* = 1, 1.6%), and Hispanic with unknown race (*n* = 2, 3.1%). No participants identified as American Indian or Alaska Native.

In terms of sex assigned at birth, most participants were assigned female (*n* = 48, 75.0%), followed by male (*n* = 13, 20.3%) and a small number who selected third gender (*n* = 1, 1.6%) or preferred not to answer (*n* = 2, 3.1%). With regards to gender, nearly half of participants identified as cisgender women (*n* = 31, 48.4%) and a smaller portion as cisgender men (*n* = 11, 17.2%). A substantial number identified as nonbinary (*n* = 16, 25.0%), with additional representation from genderqueer (*n* = 9, 14.1%), transgender men (*n* = 7, 10.9%), transgender women (*n* = 3, 5.0%), agender individuals (*n* = 2, 3.1%), and those questioning their gender (*n* = 5, 7.8%).

Regarding sexual identity, bisexual individuals comprised the largest group (*n* = 28, 43.8%), followed by those identifying as gay (*n* = 12, 18.8%), lesbian (*n* = 11, 17.2%), and queer (*n* = 7, 10.9%). Smaller groups included those who identified as asexual (*n* = 2, 3.1%), pansexual (*n* = 2, 3.1%), questioning (*n* = 1, 1.6%), and no label (*n* = 1, 1.6%).

Educational attainment varied, with most reporting some college education (*n* = 23, 35.9%) or having a 4‐year college degree (*n* = 21, 32.8%). Employment status included 13 participants (20.3%) in full‐time work, 25 (39.1%) in part‐time work, and 14 (21.9%) unemployed but seeking work. Most (*n* = 38, 59.4%) participants reported annual income less than $11,000.

Most participants (*n* = 58, 90.6%) had health insurance, including 39 (60.9%) under parental plans. Lifetime suicide attempts were reported by 28 participants (43.8%), with 14 (21.9%) indicating multiple attempts. No statistically significant differences were observed between the SPI alone and STARS groups across any variables (See Table 1).

## 5 Acceptability of the STARS Intervention

Participants reported favorable perceptions of STARS across follow‐up assessments (Table 2). On the 4‐point scale (1 = strongly disagree to 4 = strongly agree), usability items consistently averaged above 3.0, corresponding approximately to “agree.” Perceived usefulness items were moderately endorsed, with mean scores generally ranging from approximately 2.7 to 3.2, reflecting responses between “disagree” and “agree.” Although modest attenuation was observed for some perceived usefulness items at later follow‐ups, mean scores remained close to the “agree” anchor.

Participants also expressed favorable behavioral intentions. Willingness to recommend STARS to peers averaged approximately 3.15–3.22 (“agree”), and likelihood of continued use (measured on a 5‐point scale from 1 = very unlikely to 5 = very likely) ranged from 3.52 to 3.79, corresponding to responses between “neutral” and “likely.” Peer Mentor engagement items were consistently rated positively. Participants agreed that sessions were offered at convenient times (means 3.68–3.74) and reported confidence in discussing personal matters with Peer Mentors (means 3.36–3.58). Endorsement of multimedia content and goal‐setting features was moderate, with mean scores near 3.0 across follow‐up periods.

## 6 Safety Plan Use

**FIGURE 2:**
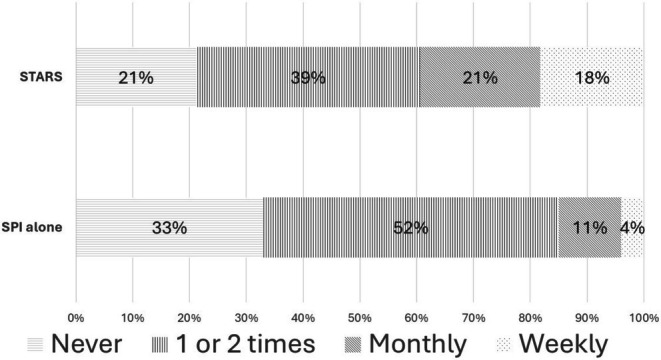
Safety Plan Use at 2 Months by Treatment Condition. Participants in the STARS condition used their Safety Plan significantly more than participants in SPI alone at 2 months (*p* < 0.05).

The repeated measures ordinal logistic regression yielded a nonsignificant treatment contrast through 6 months (*χ* ^2^ = 3.27 *p* = 0.0707; OR = 2.79, 95% CI 0.91–8.62). Participants in the STARS condition used their Safety Plan significantly more than participants in SPI alone at 2 months (*χ* ^2^ = 4.00, *p* < 0.05; OR = 2.65, 95% CI 1.01–6.79, Figure 2), such that the STARS group had greater odds of being in a higher ordinal level of Safety Plan usage than SPI alone. Safety plan use was statistically similar between SPI and STARS at 4‐ and 6‐month assessment periods (*χ* ^2^ = 2.06, *p* = 0.151; OR = 1.92, 95% CI 0.71–5.18; *χ* ^2^ = 0.46 *p* = 0.4988; OR = 1.47, 95% CI 0.52–4.15).

## 7 Suicidal Ideation

**FIGURE 3:**
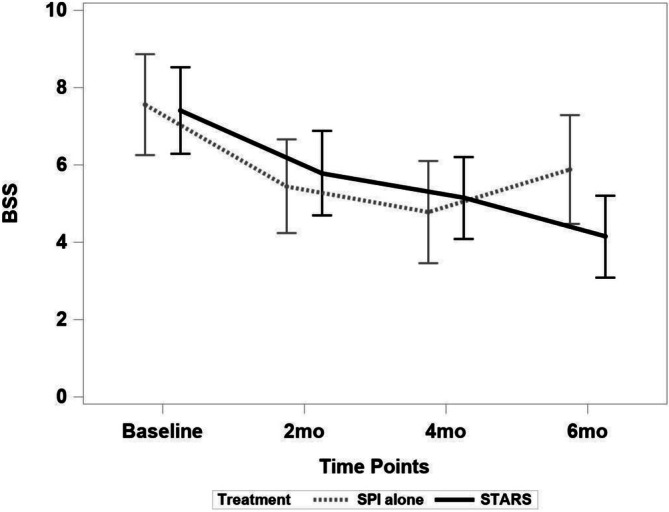
Reduction in suicidal ideation on the Beck Scale for Suicide Ideation (BSS) by Treatment Condition. Both groups experienced a significant reduction from baseline to 6‐month (*p* < 0.04) follow‐up with a nonsignificant between group difference (*p* = 0.65). BSS scores in STARS continued to improve through the entire 6‐month observation period, whereas SPI alone reported a worsening of suicidal ideation after 4 months, with a nonsignificant difference in change during the 4 to 6 month period between groups (*p* = 0.21).

On the BSS, both groups experienced a significant reduction from baseline to 6‐month follow‐up (STARS: *t* [60] = −2.78, *p* < 0.01, estimate = −2.757, SE = 0.99; SPI alone: *t* [60] = −2.13, *p* < 0.05, estimate = −2.128, SE = 0.999) with a nonsignificant between group difference *t* (60) = 0.45, *p* = 0.65, *d* = 0.12, 95% CI −0.37, 0.61. BSS scores in STARS continued to improve through the entire 6‐month observation period, whereas SPI alone reported a worsening of suicidal ideation after 4 months. Specifically, STARS reduced 0.67 (SE = 0.81) units from 4 to 6 months, whereas SPI alone worsened 0.78 (SE = 0.81) units during the 4 to 6 month period with a nonsignificant difference in change during the 4 to 6 month period between groups *t* (60) = 1.26, *p* = 0.21, *d* = 0.33, 95% CI −0.17, 0.82; Figure 3.

**FIGURE 4:**
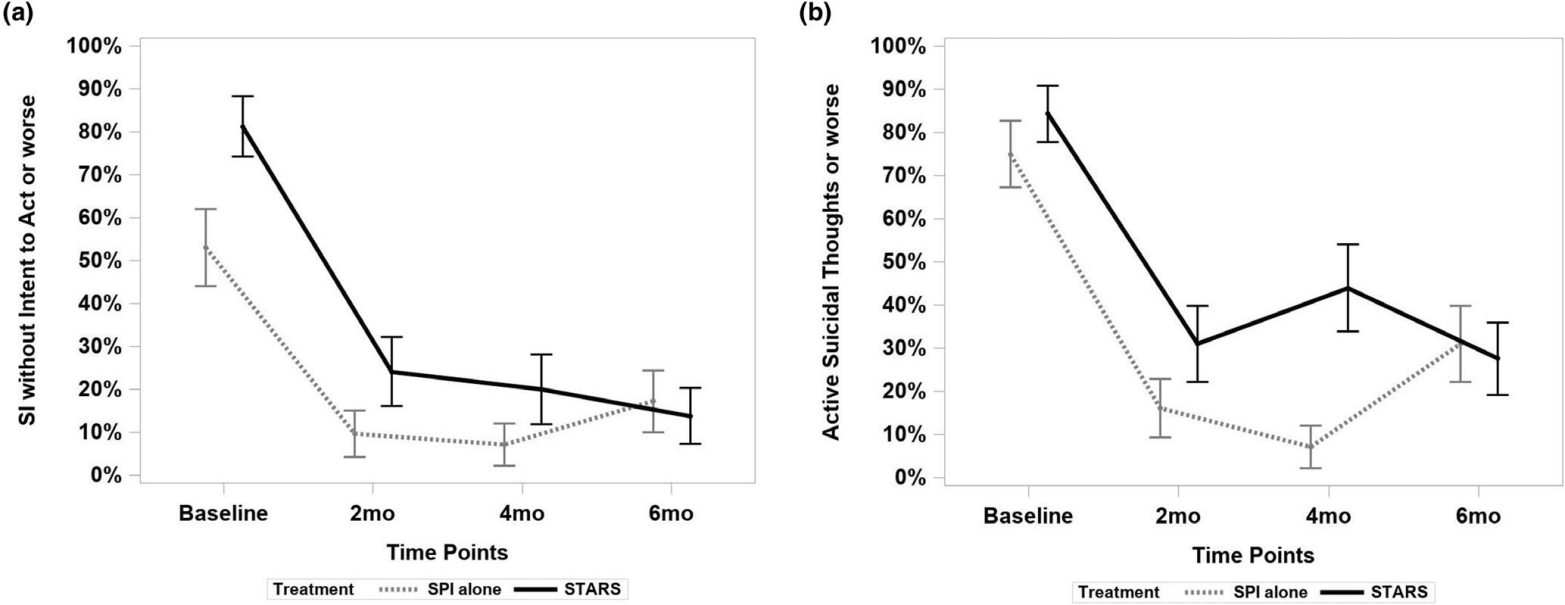
(a) Reduction in suicidal ideation without intent to act or worse by Treatment Condition. Both groups reported a significant reduction from baseline to 6‐month follow‐up (*p* < 0.001). The overall reduction in STARS was larger than SPI alone and this difference approached statistical significance (*p* = 0.058). STARS had a reduction from 4 months through 6 months, whereas SPI alone had an increase in this period, which was not a significant contrast between STARS and SPI alone (*p* = 0.25). (b) Reduction in Active Suicidal Thoughts or Worse by Treatment Condition. Both groups reported a significant reduction from baseline to 6‐month follow‐up (*p* < 0.001), though the overall reduction in STARS was nonsignificantly larger than SPI alone (*p* = 0.35). STARS had a reduction from 4 months through 6 months, whereas SPI alone had a significant increase in this period (*p* = 0.03), which was a significant contrast between STARS and SPI alone (*p* < 0.02).

On the C‐SSRS suicidal ideation subscale, when participants were categorized by severity of ideation (active suicidal ideation with a method, plan, and/or intent vs. wish to die or nonspecific active thoughts without a method, plan, or intent), both groups reported a significant reduction from baseline to 6‐month follow‐up. The overall reduction in STARS was larger (*t* [60] = −5.22, *p* < 0.0001, log‐odds estimate = −3.324, SE = 0.640) than SPI alone (*t* [60] = −3.10, *p* < 0.01, log‐odds estimate = −1.695, SE = 0.55) and this difference approached statistical significance (*t* [60] = 1.93, *p* = 0.058, OR = 0.20, 95% CI 0.04–1.06) corresponding to an 80% reduction in the odds for change in indication of active suicidal ideation with a method in mind or higher vs. not for STARS compared to SPI alone over the 6 month observation period. Similar to the BSS, continued reduction of suicidal ideation was seen in STARS participants from 4 months through 6 months (log‐odds estimate = 0.337, SE = 0.680, 4.4% reduction in prevalence), whereas there was an increase for SPI alone participants (log‐odds estimate = 0.862, SE = 0.769; 9.1% increase in prevalence; Figure 4a) which was nonsignificant (*t* [60] = 1.17, *p* = 0.25, OR = 0.30, 95% CI 0.04–2.35).

Likewise, when participants were categorized into active suicidal ideation or higher severity vs. lower severity, both groups reported a significant reduction from baseline to 6‐month follow‐up, though the overall reduction in STARS was nonsignificantly larger (*t* [60] = −4.41, *p* < 0.0001, log‐odds estimate = −2.669, SE = 0.605; 57.1% reduction in frequency) than SPI alone (*t* [60] = −3.54, *p* < 0.001, log‐odds estimate = −1.914, SE = 0.54; 44.3% reduction in prevalence), although this difference did not approach statistical significance (*t* [60] = 0.93, *p* = 0.35, OR = 0.47, 95% CI 0.10–2.30). There was a 53% reduction in the odds for change in indication of active suicidal ideation or higher vs. not for STARS participants compared to SPI alone participants over the 6‐month observation period (Figure 4b). Focusing again on the 4 month to 6 month period, STARS had a reduction from 4 months through 6 months (log‐odds estimate = 0.7074, SE = 0.547; 15.9% reduction in prevalence), whereas SPI alone had an increase (log‐odds estimate = 1.710, SE = 0.788; 23.3% increase in prevalence; Figure 4b) which was significant contrast between STARS and SPI alone (*t* [60] = 2.52, *p* = 0.014, OR = 0.09, 95% CI 0.01–0.61).

Both STARS and SPI alone groups exhibited significant reductions in suicidal ideation intensity from baseline through 6 months. The SPI alone group showed a statistically significant reduction in suicidal intensity at each follow‐up point: 2 months (Estimate = −2.50, SE = 1.13, *p* = 0.028), 4 months (Estimate = −5.97, SE = 1.14, *p* < 0.0001), and 6 months (Estimate = −5.43, SE = 1.15, *p* < 0.0001). Similarly, the STARS group demonstrated significant or marginally significant reductions at these time points: 2 months (Estimate = −2.08, SE = 1.16, *p* = 0.075), 4 months (Estimate = −2.97, SE = 1.16, *p* = 0.011), and 6 months (Estimate = −4.63, SE = 1.16, *p* < 0.0001). Although STARS showed consistent reductions across time points, none of the between‐group contrasts reached statistical significance: 2‐month difference (Estimate = −0.43, SE = 1.62, *p* = 0.791), 4‐month difference (Estimate = −3.00, SE = 1.63, *p* = 0.067), and 6‐month difference (Estimate = −0.80, SE = 1.63, *p* = 0.626).

## 8 Suicidal Behavior

Suicidal behavior at each of the follow‐up assessments is reported in Table 3. By the 6 month point, no participants in STARS reported suicidal behavior versus 2 participants in the SPI alone group, though this difference was not statistically significant (*p* = 0.16). A mixed effects logit model yielded a nonsignificant difference (*t* [60] −0.88, *p* = 0.38) with a rate of 3.6% (SE = 2.1%) for STARS compared to 6.6% (SE = 2.7%) for SPI alone.

**TABLE 3:** Suicidal behavior.

Variable	Suicidal behavior	SPI alone	STARS	*p*
2 months	Absent	28 (90.3%)	27 (96.4%)	0.3515
	Present	3 (9.7%)	1 (3.6%)	
4 months	Absent	27 (96.4%)	23 (92.0%)	0.4861
	Present	1 (3.6%)	2 (8.0%)	
6 months	Absent	27 (93.1%)	28 (100.0%)	0.1572
	Present	2 (6.9%)	0 (0%)	

## 9 Safety Plan Use in the STARS App

**FIGURE 5:**
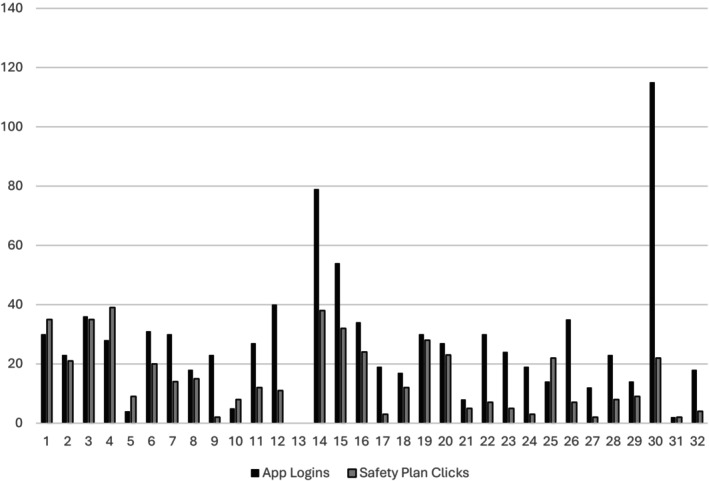
STARS logins and Navigations to Safety Plan by Participant.

Participants reported an average of 27.16 unique logins to the app (SD = 21.89) and 14.91 unique clicks on their Safety Plan in the app (SD = 11.65; Figure 5). Additionally, participants reported an average duration of 144.19 min (SD = 254.02) during all logins with an average of 12.43 min (SD = 21.52) on their Safety Plan in the app. Participants who had more frequent navigations to the Safety Plan within the STARS app reported greater reductions in suicidal ideation from baseline to 2 months (*r* = −0.471, *p* < 0.05, *n* = 26). Findings for participants with more total logins to the STARS app were similar but not significant (*r* = −0.30, *p* = 0.14, *n* = 26). Participants with longer durations of accessing their Safety Plan within the STARS app reported greater reductions in suicidal ideation from baseline to 2 months (*r* = −0.418, *p* < 0.05, *n* = 26). Findings for participants with more total duration logged in to the STARS app were similar but not significant (*r* = −0.32, *p* = 0.11, *n* = 26).

## 10 Discussion

Pilot trial findings support the preliminary acceptability, safety, and potential efficacy of the STARS intervention as a tailored, multicomponent approach to suicide prevention for EA‐SGM youth. Developed to address structural and interpersonal barriers to Safety Plan use, STARS combines the evidence‐based SPI with mobile app functionality and peer mentorship. This hybrid approach addresses known barriers to SPI implementation and may promote sustained engagement.

STARS participants reported significantly more frequent Safety Plan use at 2‐months, suggesting that digital tools and peer support may enhance early engagement. This pattern aligns with previous literature emphasizing the importance of accessibility and reinforcement for Safety Plan use (Kayman et al. 2015). Group differences were not statistically significant at subsequent timepoints.

We observed high acceptability ratings across follow‐ups, indicating participants found STARS usable and relevant, key factors associated with uptake and sustainment. Acceptability indicators were generally in line with mobile apps for suicide prevention in other populations, including Veterans (Primack et al. 2022) and adolescents (McManama O'Brien et al. 2017). Peer mentoring was also rated positively (Brown et al. 2023; Tran et al. 2025). These findings align with previous research indicating that peer‐based support can enhance intervention uptake and engagement (Kaufman et al. 2022). Unlike prior studies of peer mentoring delivered via telehealth, STARS integrated app‐based support between sessions. Future research should examine whether adaptive digital nudges or booster peer mentoring sessions beyond the initial 6‐week period may sustain consistent use of the Safety Plan over time and should explore the effect of dismantling effects of the STARS app from peer mentorship on core outcomes.

The consistency of effects among STARS participants suggests potential clinical relevance. Both groups reported significant reductions in suicidal ideation over the 6‐month follow‐up period, though STARS participants exhibited greater overall reductions in active suicidal ideation, particularly from months 4 to 6, when suicidal ideation in the SPI alone group slightly increased. These clinically meaningful, albeit modest, effects support the added value of digital and peer‐based enhancements to SPI. These findings are especially promising considering implementation science priorities that emphasize real‐world feasibility and scalability.

In fact, exploratory analyses suggested that app engagement was associated with reductions in suicidal ideation among participants in the STARS group. While these analyses are preliminary, they point to engagement with intervention content as a potential mechanism of action. This supports our hypothesis that greater engagement with the STARS app would be associated with improved outcomes. It is also possible that peer mentors played a role in reinforcing app engagement, helping participants overcome motivational or emotional barriers to using their Safety Plan, suggesting a synergistic effect between digital and relational components.

## 11 Limitations and Future Directions

This study has several limitations. First, as a pilot trial, the primary goal of this study was evaluating feasibility and acceptability, and it is possible that statistical differences in clinical outcomes may not emerge in a fully powered clinical trial. Second, recruitment from a single metropolitan area may limit generalizability, and self‐selection bias cannot be ruled out. Future work should also explore the feasibility of embedding STARS into real‐world service settings, such as campus counseling centers, primary care practices, emergency departments, or telehealth platforms. Embedding the intervention in settings where EA‐SGM youth already seek care could enhance scalability and integration into existing mental health service systems. Third, reliance on self‐report may introduce bias, though validated scales were used to mitigate this concern. Fourth, because STARS combined app and peer mentoring, we cannot disentangle their individual effects. Fifth, future evaluations should include an evaluation of the frequency of suicidal crises to establish the number of times when a participant should use their Safety Plan in a given week. Sixth, while our sensitivity analyses through multiple imputation did not find any evidence of potential bias attributable to our missing data mechanism, missing data poses an analytic limitation. Finally, given the small sample size of our pilot trial, rates of suicidal behavior were very low, precluding the ability to detect group differences. Future research should explore the efficacy of STARS in a sample recruited in acute suicidal crises or with a history of suicide attempts, which might increase the ability to detect differences in suicide attempts with adequate power.

## 12 Conclusion

In conclusion, this pilot trial offers initial evidence supporting the feasibility, acceptability, and potential clinical benefits of the STARS intervention for EA‐SGM youth at risk for suicide. STARS may help bridge gaps in LGBTQ+ affirming suicide prevention. Continued refinement of app features and integration with existing systems may enhance its impact, as might evaluation of varying doses and delivery formats for peer mentorship. STARS addresses key implementation gaps and presents a promising direction for equitable mental health care.

## Author Contributions

**Amanda Arcomano:** writing – review and editing, project administration, data curation. **Robert Gallop:** software, formal analysis, writing – review and editing, visualization. **Jennifer T. Tran:** writing – review and editing, methodology, resources, data curation, project administration. **Lily A. Brown:** conceptualization, investigation, funding acquisition, methodology, project administration, supervision, data curation, resources, writing – original draft. **James R. Wolfe:** writing – review and editing, supervision, project administration. **Yiqin Zhu:** writing – review and editing, data curation, project administration. **Danielle Mowery:** writing – review and editing, investigation, methodology, funding acquisition, conceptualization. **Alexander Azat O'Connor:** writing – review and editing, project administration, data curation. **José A. Bauermeister:** writing – review and editing, writing – original draft, conceptualization, investigation, funding acquisition, methodology, validation, project administration, data curation, supervision, resources. **Maria A. Oquendo:** conceptualization, investigation, funding acquisition, writing – review and editing. **Gregory K. Brown:** conceptualization, writing – review and editing, investigation, funding acquisition. **Jennifer Ben Nathan:** writing – review and editing, project administration, data curation. **Marin M. Kautz:** writing – review and editing, data curation, project administration, methodology. **Lindiwe Mayinja:** writing – review and editing, project administration, data curation. **Jessica L. Webster:** resources, project administration, writing – review and editing. **David S. Mandell:** writing – review and editing, supervision, conceptualization, investigation, funding acquisition.

## Funding

This work was supported by the National Institutes of Health (P50 MH127511).

## Ethics Statement

This study was approved by the University of Pennsylvania IRB (protocol 849500).

## Consent

Participants provided informed consent to participate.

## Conflicts of Interest

G.K.B. and M.A.O. receive royalties for the commercial use of the Columbia–Suicide Severity Rating Scale from the Research Foundation of Mental Hygiene Inc. MO serves as an advisor to Alkermes, Mind Medicine, St George's University, and Fundación Jimenez Díaz. Her family owned stock in Bristol Myers Squibb.

## Data Availability

The data that support the findings of this study are openly available in NIMH Data Archive at https://nda.nih.gov/edit_collection.html?id=4115, reference number C4115.
